# Early Results of a New Rotating Hinge Knee Implant

**DOI:** 10.1155/2014/948520

**Published:** 2014-06-25

**Authors:** Alexander Giurea, Hans-Joachim Neuhaus, Rolf Miehlke, Reinhard Schuh, Richard Lass, Bernd Kubista, Reinhard Windhager

**Affiliations:** ^1^Department of Orthopaedics, Vienna General Hospital, Medical University of Vienna, Waehringer Guertel 18-20, 1090 Vienna, Austria; ^2^Department of Traumatology and Orthopaedics, St. Vincenz Hospital Am Stein 24, 58706 Menden, Germany; ^3^The Rhine-Main Center of Joint Diseases, Wilhelmstraße 30, 65183 Wiesbaden, Germany

## Abstract

*Background*. Indication for rotating hinge (RH) total knee arthroplasty (TKA) includes primary and revision cases, with contradictory results. The aim of this study was to report prospective early results of a new modular rotating hinge TKA (EnduRo). For this implant several new design features and a new bearing material (carbon-fiber reinforced poly-ether-ether-ketone) have been developed. Furthermore, we tried to establish a new classification of failure modes for revision TKA. *Methods*. 152 EnduRo rotating-hinge prostheses were implanted in two centers. In 90 patients a primary implantation has been performed and 62 patients were revision cases. Knee Society Score (KSS), Western Ontario and McMaster Osteoarthritis Index (WOMAC), Oxford Knee Score (OKS), and Range of motion (ROM) were assessed before surgery, 3 months postoperatively, 12 months postoperatively, and annually thereafter. We defined 3 types of complications: Type 1, infection; type 2, periprosthetic complications; type 3, implant failures. *Results*. KSS, WOMAC, OKS, and ROM revealed significant improvements between the preoperative and the follow-up investigations. There were 14 complications (9.2%) leading to revision surgery, predominantly type 2. *Conclusion*. Our study shows excellent clinical results of the EnduRo TKA. Furthermore, no premature material failure or unusual biological response to the new bearing material could be detected.

## 1. Introduction

The rate of revision total knee arthroplasties (TKAs) is steadily increasing [1]. The main reasons are infection, aseptic loosening, instability, and microdebris-related osteolysis. With increasing numbers of severe cases with bone loss, instability, and comminuted distal femoral fractures, a more constrained prosthesis such as a rotating hinge TKA is often warranted [2, 3].

Hinged prostheses were first designed and used for reconstruction after wide resection of neoplasms around the knee joint. The initial joint mechanism consisted in a fixed hinge with no rotational motion. This led to high rates of early loosening, osteolysis, and excessive wear due to the highly restricted biomechanics [4–6]. A second generation modified several aspects (rotational axis with a stop, new design of the patellofemoral joint to facilitate patella tracking, use of a metallic tibial baseplate to reduce polyethylene wear, and improvements in the stem design to facilitate osteointegration). In order to avoid torsional stresses on the bone implant interface flexing and rotating models have been introduced. This design aims to avoid early aseptic loosening of the prosthesis [7–11].

There are several indications for the use of rotating hinge knees (RHK). In severe primary osteoarthritis (excessive varus-/valgus deformities, Charcot knees, and rheumatoid arthritis) as well as in revision-cases of TKA rotating hinge prostheses are necessary to compensate for ligamentous instability as well as for severe bone loss [2, 4, 12, 13].

Several authors have studied the results of rotating hinge TKA for various types of prosthesis. However, their conclusions are contradictory. Certain authors seem to consider such devices to be useful mainly in salvage procedures after numerous failed revisions, whereas others have described encouraging outcomes [6, 14–16]. There are only a few prospective studies with a follow-up of more than two years of rotating hinge TKA in nontumour cases.

The EnduRo prosthesis (Aesculap AG, Tuttlingen, Germany) represents a new modular rotating hinge design. It is characterized by transmission of force from the femoral component to the tibial component via the polyethylene insert with high contact area. Therefore, the hinge is not primary weight bearing. In order to address certain anatomical situations it offers an offset option and wedges for the tibial and the femoral components. Additionally, it contains carbon-fibre reinforced poly-ether-ether-ketone (CRF-PEEK) flanges and bushings firstly used as a bearing material in TKA [17]. Biotribological in vitro studies showed decreased wear for this implant [17]. Furthermore an in vivo murine model showed similiar biological response to CFR-PEEK, if compared to polyethylene debris particles [18].

The aim of the present clinical study was to investigate prospectively the functional and radiographic results of the EnduRo rotating hinge TKA in a short-term clinical follow-up with regard to the newly introduced bearing material. Also, a new classification of failure modes of rotating hinge TKA has been established in order to gain better information about implant survivorship.

## 2. Patients and Methods

### 2.1. Study Population

The study received ethical approval from the regional institutional review board (Ref. number 703/2009). An informed consent was given by every patient. Our study is a prospective clinical two-center study. Between November 2008 and December 2012, 152 EnduRo rotating hinge prostheses were implanted at two different orthopaedic centers center 1: (*n* = 59) and center 2: (*n* = 93). Mean age at the time of implantation was 72.3 years (SD 9.3; range 48.0–90.0). Mean body weight was 85.0 kg and height 168.4 cm resulting in an average body mass index (BMI) of 29.8 (SD 6.3, range 15.0–50.0). There were 47 male and 105 female patients. In 90 patients a primary implantation has been performed and 62 patients were revision cases where the EnduRo RHK was used. In center 1 the ratio of primary to revision was 44% to 56% and in center 2 69% of the patients received the device as a primary and 31% as a revision implant. Demographic parameters are illustrated in Table 1. Data collection included demographic information, a complete medical history, and the indication for primary or revision TKA.

Indications for primary arthroplasty with the EnduRo system included severe varus or valgus osteoarthritis (more than 20°) with substantial ligamentous instability, rheumatoid arthritis with ligament laxity, and posttraumatic and postinfectious gonarthrosis. Indications for revision arthroplasty included septic or aseptic loosening as well as instability after primary total knee arthroplasty including flexion extension gap mismatch. Table 1 shows the indications of the patients of the present study.

The diagnosis of septic or aseptic loosening, respectively, was based on a thorough clinical, radiographic, and microbiological examination combined with laboratory studies including leukocyte counts, sedimentation rate, and C-reactive protein. The preoperative diagnoses have been confirmed by histologic analysis and bacterial cultures.

### 2.2. Type of Prosthesis

The EnduRo prosthesis was used in all patients. It is a modular rotating hinge prosthesis with primary transmission of force from the femur via the polyethylene (PE) insert to the tibial part of the prosthesis. The contact area between the metal and the PE surface is considerably high with a minimum of 800 mm^2^ during the entire range of motion (ROM). The axis is not primarily weight bearing but provides stability of the joint in case of severe coronal or sagittal instability. The axes are embedded in bushings and flanges made of PEEK-Optima LT1 (Invibio Ltd., Thornton-Cleveleys, UK) with a carbon-fiber reinforcement containing 30% polyacrylonitrile- (PAN-) based carbon fibers (CFR-PEEK LT1 CA 30) firstly used as a bearing articulation in TKA in order to minimize wear and creep [17, 19]. The prosthesis is designed for a ROM from 3° of hyperextension to 135° of flexion. Augments are available as tibial and femoral wedges in different heights. The prosthesis provides an offset option for femoral and tibial stems. Fixation of the stem is available as cemented or cementless, whereas the epiphyseal fixation of the prosthesis is always cemented.

### 2.3. Surgical Procedures

All 152 surgical procedures have been carried out through a medial parapatellar arthrotomy. Patients were given a perioperative antibiotic prophylaxis with Cefazolin (3 × 2 g) or Clindamycin (3 × 600 mg). In case of two-stage revision arthroplasty due to septic complication antibiotic therapy was chosen according to microbiologic cultures and sensitivity and given for at least 6 weeks between stages and for 6 weeks (range 6 to 13 weeks) after the second stage procedure using the EnduRo RHK. Thrombotic prophylaxis was used with low-molecular weight heparin (40 mg–60 mg/day) starting 12 hours before surgery continuing for 6 weeks postoperatively. A tourniquet was used before osteotomies have been carried out and released after implant fixation. All implants were implanted in the hybrid technique with cemented femoral and tibial epiphyseal fixation and uncemented stems. Gentamycin containing cement (Palacos R + G, Heraeus, Hanau, Germany) and vacuum cementing technique were used. The patella was routinely resurfaced and a lateral release was undertaken when necessary to achieve satisfactory patellar tracking. The postoperative management was similar for all patients with the use of crutches or a walker as long as needed and with partial to full weight bearing as tolerated.

**Figure 1:**
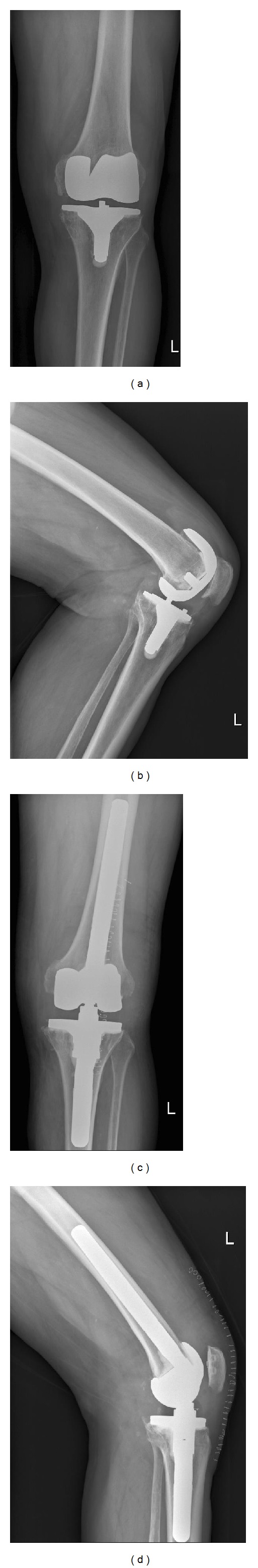
(a) Radiographs of aseptic loosened TKA in 77-year-old male—ap view. (b) Radiographs of aseptic loosened TKA in 77-year-old male—lateral view. (c) Postoperative X-rays after one stage revision with EnduRo RHK—ap view. (d) Postoperative X-rays after one stage revision with EnduRo RHK—lateral view.

**Figure 2:**
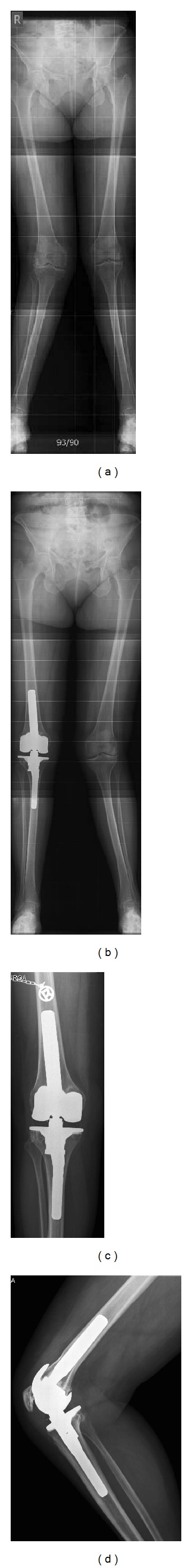
(a) Preoperative long standing X-ray of 73-year-old female with 22° valgus deformity. (b) Postoperative longstanding X-ray. (c) Detail X-ray—ap view. (d) Detail X-ray—lateral view.

Primary procedures were carried out in 90 and revision procedures were carried out in 62 knees, whereas in center 1 the ratio primary/revision was 26/33 and in center 2 it was 64/29 (Figures 1 and 2).

### 2.4. Clinical Outcome and Patients Satisfaction Measures

Clinical outcome of patients was assessed using the Knee Society Score (KSS clinical, function) [20], the Western Ontario and McMaster Osteoarthritis Index (WOMAC) [21], and the Oxford Knee Score, a 12-item patient-reported score [22]. Range of motion (ROM) was measured passively with a goniometer with the patient in supine position. Examinations were carried out before surgery, 3 months postoperatively, 12 months postoperatively, and annually thereafter.

### 2.5. Radiographic Analysis

Standard anteroposterior and lateral digital projection radiographs of the knees were obtained using Siemens Aristos digital radiographic workplace (Siemens, Erlangen, Germany). All imaging was performed at 57–63 kV and exposure values of 8–12 mAS. (Figures 1 and 2). Evaluation was done by 2 independent observers using standard pacs system (Impax Agfa health care, Mortsel, Belgium). Interobserver reliability was assessed by comparing radiologic reports. Radiographic analysis included assessment of alignment, signs of loosening such as component migration, radiolucent lines of >2 mm, presence of cement fracture or prosthetic/peri-prosthetic fracture, osteolysis, and wear, according to the appropriateness criteria of the American College of Radiology.

### 2.6. Complications

Complications were defined as complications leading to revision surgery of the involved knee. We defined 3 types of complications: type 1, infection; type 2, periprosthetic complications such as periprosthetic fracture, extensor mechanism failure, patella problems, and wound healing disturbances; type 3, implant complications such as aseptic loosening, wear, implant failure (fracture of axis, bushings, and stem), and instability. Type 1 complications (infections) and type 2 complications (periprosthetic complications) occur with no or little impact of the prosthesis on complications, whereas type 3 complications give information about survivorship of the implant.

### 2.7. Statistical Analysis

Two-sided tests were used in order to determine statistical significance. The level for statistical significance was set to 0.05 for all tests. Student's *t* test and analysis of variance (ANOVAs) were performed for continuous variables (age, BMI, hospital stay, and operation time).

Chi-squared tests were used for categorical variables (gender, diagnosis, and complication rate). Survival rates were estimated by Kaplan-Meier method and analyzed by Cox regression. Dunnett's test was used for comparisons of postoperative scores to the preoperative score to control the first type error. All regression analyses were adjusted for age, gender, BMI, and preoperative values. Statistical analysis was performed using SAS 9.2 (SAS Inc. Chicago, IL) and STATA 12.1 (StataCorp, College Station, TX).

## 3. Results

### 3.1. Clinical Parameters

Results are presented in Table 2. The outcome scores revealed statistically significant improvements between the preoperative and the follow-up investigations. The WOMAC score was 4.85 (SD 2.06) presurgically and 1.86 (SD 1.60) at 3 months, 1.66 (SD 1.88) at 12 months, and 1.62 (SD 1.67) at 24 months after surgery. The Oxford Knee Score improved from 19.1 (SD 8.1) to 33.0 (SD 8.2), 34.6 (SD 9.0), and 35.8 (SD 8.7), respectively. The Knee Society Score (KSS) improved from 26.1 (SD 16.0) preoperatively to 84.0 (SD 14.6), 85.2 (SD 14.6), and 89.0 (SD 14.7). Knee Society Score function (KSSf) was 38.6 (SD 23.8) prior to surgery and 57.8 (SD 23.2), 64.8 (SD 20.3), and 65.6 (SD 21.3) at the respective timepoints. ROM increased from 94.4° (SD 32.3°) before surgery to 110.1° (SD 14.0°), 114.1° (SD 12.2°), and 119.0° (11.5°). The clinical parameters for primary and revision cases are shown in detail in Table 2.

There was a significant influence of the indication for surgery (primary or revision) on the outcome. We found significant better scores preoperatively as well as postoperatively when the procedure was carried out as primary arthroplasty (Table 3).

There was significant impact of gender (*P* = 0.043), diagnosis (*P* = 0.006), and timepoint of followup (*P* = 0.011) on the postoperative improvement in terms of KSS clinical score. Female patients as well as revision procedures showed inferior results (Table 4(a)). For KSS function preoperative score (*P* < 0.0001), gender (*P* = 0.018), and timepoint of followup (*P* = 0.011) had significant influence (Table 4(b)). There was no impact of center and age on KSS.

### 3.2. Radiologic Results

In our cohort no interobserver variability could be detected. In patients with type 2 (periprosthetic complications) and type 3 complications (implant failure) radiologic irregularities were reported by both observers. In all other patients there were no signs of radiologic complications reported by both observers.

### 3.3. Complications

There were 14 complications (9.2%) leading to revision surgery.

*Type 1.* Deep infections occurred in 5 cases (4 patients; 3.3%) and were treated by two-stage revision procedure with implant removal, spacer implantation, and reimplantation of an EnduRo RHK (4 cases) and 1 tumor prosthesis (distal femoral replacement, Modular Rotating Hinge, Stryker, Warsaw, USA).

*Type 2.* Complications occurred in 6 patients (3.9%): two periprosthetic fractures have been treated with locking plate osteosynthesis; 2 extensor mechanism ruptures and 2 patella dislocations have been treated by extensor mechanism reconstruction and postoperative immobilisation for 2–6 weeks.

*Type 3.* Prosthesis complications were seen in 3 patients (2.0%): 2 aseptic loosening of the femur were treated by one stage revision with revision of the femoral part in hybrid technique in one and cemented in the other. One loose tibial rotational axis locking screw was revised by a new fixating screw.

Regression analysis revealed a higher risk of reoperation for revision cases than for primary implantations (HR: 6.00, 95% confidence interval (CI): 1.76–20.47, *P* = 0.004). In revision cases the subgroup of septic loosening showed the highest tendency but no significance for reoperation (HR: 3.14, CI: 0.85–11.54, *P* = 0.08). Furthermore high baseline WOMAC score was associated with a decreased risk for reoperation (HR: 0.656, CI: 0.44–0.96, *P* = 0.037). There was no impact of age, gender, BMI, or center on revision rate.

### 3.4. Survival Analysis

**Figure 3:**
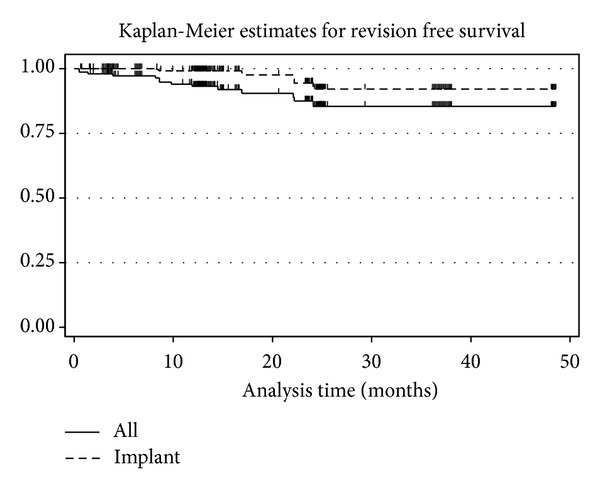
Kaplan-Meier survival curves; total and implant survival.

The overall survivorship with revision for any reason as endpoint was 85.4% (CI: 75.6–91.5) at 2 years. The implant associated survivorship was 92.1% (CI: 81.5–96.8). (Figure 3).

## 4. Discussion

The number of primary and revision TKA is increasing. Many knee surgeons recommend in case of revision surgery designs with the lowest level of constraint possible [6]. Problems with conventional constrained TKA were early aseptic loosening and implant failure due to highly restricted biomechanics. Traditionally, transmission of force was conducted by the hinge axis of the implant. The design of the implant presented in this study addresses this problem by shifting force transmission through the condylar area with additional rotational motion around the tibial axis. Therefore, decreased shear stress is applied to the bone-implant interface. In this prospective study we present early results of a new rotating hinge TKA implant in a fairly high number of patients. The results of the present study reveal favourable results in terms of functional improvement and ROM. The overall survival rate was 85.4%. Patients, who received the implant in revision surgery, experienced a six times higher risk of reoperation than patients after primary implantation. We introduced a new classification for TKA complications to receive more information about prosthesis failure. Type 1 complications (infections) occur unrelated to the prosthesis. In type 2 complications (periprosthetic complications) surgical technique and tissue quality seem to play a certain role rather than the implant design. Type 3 complications (implant failures) give information about survivorship of the prosthesis. The most common type of complication was periprosthetic complications (type 2). They occurred in 3.9% of our cases.

There are some limitations associated with the present study. First, there is a relatively short follow-up period presented. However, infection, aseptic loosening, and mechanical failure occur most frequently within the first two years after implantation [1, 3, 6, 14]. Therefore, we do think that preliminary results provide reasonable information to the academic community especially when a new implant with clinically unproven design features and CFR-PEEK as a new bearing material is used. Second, we followed only 62 patients at two years, as the other patients did not reach the two-year followup yet. This fact was considered in our statistics and 62 patients represent an acceptable high number especially for rotating hinge studies.

The results of the present study reveal an improvement of KSS from 26.1 to 85.2 and 89.0 one and two years postsurgically, respectively, and of KSS function (*f*) from 38.6. to 64.8 and 65.6. There was only a slight increase between one and two years postoperatively. Westrich et al. [23] reported in 24 rotating hinge TKAs (9 primary/15 revisions) at a mean followup of 33 months 83 points for KSS and 45 points for KSSf. Whereas KSS corresponds to the results of the present study, KSSf of the present study is superior to the study of Westrich et al. [23]. This may be due to the improved biomechanics of the implant used in the patients of our study. In 2007 Pour et al. [2] found in 44 rotating hinge TKAs (17 primary/27 revisions) at an average followup of 50.4 months a KSS of 73.5 and a KSSf of 43. Again in this study the KSSf presented is inferior to ours. In a recent study Hernández-Vaquero and Sandoval-García [24] found in 26 rotating hinge TKAs (5 primary/21 revisions) 46 months after surgery a KSS of 77 and a KSSf of 51. In a recent study, Yang et al. [11] investigated 50 primary rotating hinge TKAs with a mean followup of 15 years. They found an increase in KSS from 38 to 73 and for KSSf from 36 to 47.

We found a total of 9.2% of complications requiring revision surgery in the patients of the present study. Böhm and Holy [25] reported in 422 rotating hinge TKAs 3.8% infections, 0.7% aseptic loosening, and 0.2% patellar complications at a mean followup of 6 years. Westrich et al. [23] found no aseptic loosening in 24 rotating hinge TKAs after 33 months but 8.3% periprosthetic fractures. Hernández-Vaquero and Sandoval-García [24] found in 2010 in 26 rotating hinge TKAs 3.8% periprosthetic fractures and 7.7% infections.

Recently, van Kempen et al. [26] reported in a prospective study results of 150 revision arthroplasties using a modern semiconstrained implant design. Theoretically, we would expect that functional outcome and complication rate were superior with a less constraint design compared to the results of the present study. In fact, they found a KSS of 76 and a KSSf of 61 points, respectively, at 24 months after surgery. ROM improved from 93° preoperatively to 107° at followup. In their study, 22 complications occured leading to revision surgery resulting in a complication rate of 14.6%. Thus, results are not superior to the results of the present study with a more constrained implant design.

We do think that rotating hinge implants facilitate ROM in severe primary and revision cases and are favourable for all indications of revision TKA. Our results show an increase of ROM from 94° preoperatively to 119° 24 months after surgery. We found 110° ROM 3 months postoperatively with significant increase after 12 and 24 months. There was no revision due to TKA instability in our study, which often occurs within the first two years after TKA.

Biotribologic studies have shown in vitro promising low rates of wear and debris for the EnduRo prosthesis [17]. We were interested if these findings will correlate in clinical outcome. In our in vivo study we found no noticeable signs of biological response to the new CFR-PEEK PAN bearing material. This macroscopic clinical view is also supported by cell culture experiments on L929 and U937 cell lines [27], showing that CFR-PEEK PAN wear particles had no cytotoxic effects and would possibly not cause adverse tissue reactions in vivo. Injecting CFR-PEEK PAN particles intra-articular in a mice model, a similar biological response was found if compared to UHMWPE [18]. A clinical study investigating retrieved tissue of a CFR-PEEK pitch acetabular liner in hip replacement showed a grey synovium due to the CFR-PEEK wear particles but without any serious inflammatory reaction [28].

## 5. Conclusion

The preliminary results of the present study reveal that the EnduRo rotating hinge TKA yields good clinical, functional, and radiologic results at a short-term followup. Functional results appear to be superior to those reported for other rotating hinge TKA implants. We do believe that this is due to improvements of implant biomechanics and biomaterials. When applied in revision surgery, there is a six times higher risk for reoperation than in primary use. Long-term results have to be presented in order to draw definitive conclusions regarding the survival rate.

## Figures and Tables

**Table 1:** Demographic and indication parameters of 152 study cases.

Age (mean, range)	72.3; 48–90
Body mass index (mean, range)	29.8; 15–50
Male/female	47/105

Primary	**90 (59.2%) **
Severe primary (varus/valgus/instable)	85 (55.9%)
Rheumatoid arthritis	1 (0.7%)
Posttraumatic arthritis	3 (1.9%)
Postinfectious arthritis	1 (0.7%)

Revision	**62 (40.8%) **
Septic loosening	19 (12.5%)
Aseptic loosening	20 (13.2%)
Instability after primary TKA	23 (15.1%)

**Table 2:** Outcome parameter total and in primary and revision cases.

	Preoperative	3 months	12 months	24 months
WOMAC (mean; STD)	**4.9; 2.1**	**1.9; 1.6 **	**1.7; 1.9 **	**1.6; 1.7 **
Primary (mean; STD)	4.5; 2.0	1.6; 1.4	1.3; 1.5	1.2; 1.2
Revision (mean; STD)	5.3; 2.1	2.3; 1.8	2.3; 2.3	2.3; 2.0

Oxford Knee Score (mean; STD)	**19.1; 8.1 **	**33.0; 8.2 **	**34.6; 9.0 **	**35.8; 8.7 **
Primary (mean; STD)	20.4; 7.8	34.9; 7.8	36.4; 8.2	39.4; 6.1
Revision (mean; STD)	17.2; 8.3	29.9; 8.1	31.7; 10.0	30.4; 9.7

KSS clinical (mean; STD)	**26.1; 16.0 **	**84.0; 14.6 **	**85.2; 14.6 **	**89.0; 14.7 **
Primary (mean; STD)	22.5; 14.3	86.0; 13.7	87.7; 12.7	94.1; 8.0
Revision (mean; STD)	31.5; 17.0	80.7; 15.5	80.9; 16.5	81.4; 18.9

KSS function (mean; STD)	**38.6; 23.8 **	**57.8; 23.2 **	**64.8; 20.3 **	**65.6; 21.3 **
Primary (mean; STD)	43.1; 24.3	61.2; 22.5	66.5; 19.7	72.1; 14.0
Revision (mean; STD)	32.2; 21.8	52.3; 23.6	61.9; 21.1	56.0; 26.4

ROM (mean; STD)	**94.4; 32.3 **	**110.1; 14.0 **	**114.2; 12.2 **	**119.0; 11.5 **
Primary (mean; STD)	103.3; 21.2	112.8; 11.2	115.7; 11.4	120.3; 9.1
Revision (mean; STD)	81.2; 40.7	105.8; 17.0	111.4; 12.9	117.2; 14.3

**Table 3:** *P* values for outcome scores in comparison of primary and revision procedures.

	Preoperative	3 months	12 months	24 months
WOMAC	0.0308	0.0114	0.0083	0.0084
Oxford Knee Score	0.0182	0.0007	0.0072	<0.0001
KSS clinical	0.0006	0.0470	0.0117	0.0006
KSS function	0.0053	0.0368	0.2386	0.0027
ROM	<0.0001	0.0047	0.0777	0.3056

**Table:** (a) KSS clinical

Effect	*P* value
Kss1_k0	0.5238
Age	0.5574
Gender	0.0427
DiagKat2	0.0006
FUKat	0.0109
Center	0.8122

**Table:** (b) KSS_function

Effect	*P* value
kss_f0	<0.0001
Age	0.2225
Gender	0.0183
DiagKat2	0.1460
FUKat	0.0007
Center	0.1456
